# Membrane properties of hydroxycholesterols related to the brain cholesterol metabolism

**DOI:** 10.3762/bjoc.13.71

**Published:** 2017-04-18

**Authors:** Malte Hilsch, Ivan Haralampiev, Peter Müller, Daniel Huster, Holger A Scheidt

**Affiliations:** 1 Department of Biology, Humboldt University Berlin, Invalidenstraße 43, D-10115 Berlin, Germany; 2 Institute for Medical Physics and Biophysics, Leipzig University, Härtelstr. 16–18, D-04107 Leipzig, Germany

**Keywords:** cholesterol, fluorescence, hydroxycholesterol, membrane structure, NMR

## Abstract

Compared to cholesterol, hydroxycholesterols contain an additional hydroxy group in the alkyl chain and are able to efficiently cross the brain–blood barrier. Therefore, they are responsible for the sterol transfer between brain and circulation. The current study compares the membrane properties of several hydroxycholesterols with those of cholesterol using ^2^H NMR spectroscopy, a membrane permeability assay, and fluorescence microscopy experiments. It is shown that hydroxycholesterols do not exert the unique impact on membrane properties characteristic for cholesterol with regard to the influence on lipid chain order, membrane permeability and formation of lateral domains.

## Introduction

Cholesterol is a major component of mammalian cell membranes with various biological functions. It plays a key role in maintaining the membrane’s barrier function by increasing the bilayer packing density through condensing the phospholipids. Furthermore, cholesterol is an important player in the dynamic domain structure of the plasma membrane and the formation of lateral lipid domains with relevance to membrane protein function, protein trafficking, and intramembrane proteolysis [1–3]. A large amount of cholesterol in the human body is located in the brain, where it constitutes an integral part of myelin membranes acting as electrical insulators [4]. Cholesterol is also a major component of the plasma membranes of astrocytes and neurons [5]. The tight control of the cholesterol concentration and homeostasis is of paramount importance for the functions of all cells of the body but particularly for the brain. More so as the rate of cholesterol accumulation and synthesis is subject to subtle alterations over the lifetime of a human being [4,6].

**Figure 1:**
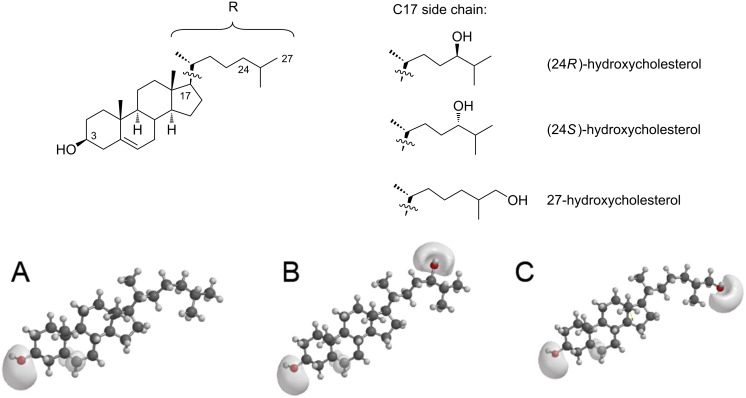
Top: Chemical structures of cholesterol and hydroxycholesterols with selected numbering for the carbon atoms. The investigated hydroxycholesterols are modified with an OH-group bound to the carbon in position 24 (either in *R*- or *S*-configuration) or in position 27, respectively. Bottom: Molecular models and isosurfaces of the electrostatic potential of cholesterol (A), (24*R*)-hydroxycholesterol (B) and 27-hydroxycholesterol (C).

Insoluble cholesterol is transported in the blood in small lipoprotein particles of varying density and the rate, at which cholesterol crosses the lipid membrane, is extremely low [7]. To overcome the blood–brain barrier, nature has developed efficient mechanisms to convert cholesterol into metabolites, which can easily diffuse into the brain. These metabolites are primarily the oxysterols (24*S*)-hydroxycholesterol (24*S*-HC) for the transport from the brain to the bloodstream and 27-hydroxycholesterol (27-HC) for the transport in the opposite direction [4,6,8]. These molecules are modified by a hydroxy group at the alkyl chain end of the cholesterol molecule, introducing a second polar center to the hydrophobic tetracyclic ring system in addition to the OH moiety of cholesterol (Figure 1). This putatively simple modification is responsible for the better membrane penetrability (see below, [7]). A very interesting finding is that the plasma level of 24*S*-HC, which is nearly exclusively produced in the brain [6], can be used as a marker for neurodegenerative and neurological diseases including Alzheimer’s diseases [4,6,8–9].

The biophysical properties of cholesterol and their influence on lipid membranes have been widely investigated as a basis for the understanding of the cell biological importance of the molecule. It has been shown that the membrane properties of cholesterol are extremely well adapted to exert a very specific influence on the other membrane constituents, resulting in highly characteristic effects on the packing and lateral organization of the membrane lipids and proteins [1,10–13]. Even very small alterations in the molecular structure of cholesterol cause large differences in the interaction with phospholipids and its membrane’s barrier function. Interestingly, not only modifications in the tetracyclic sterol ring system lead to pronounced changes of the molecular membrane architecture and of the interactions between the respective sterol and membrane lipids and proteins [14–17], but also the *iso*-branched side chain of cholesterol has an important impact on the membrane properties [18–20].

For the hydroxysterols 24*S*-HC and 27-HC, little experimental data on their influence on membrane properties are available. So far, only slightly altered lipid mobility was observed in the presence of both hydroxysterols using fluorescence techniques [21]. Also, a decreased but still significant effect of the hydroxysterols on lipid condensation compared to native cholesterol was found in molecular dynamics simulations, which is probably caused by an increased tilt angle of the sterols to the membrane normal [8,21]. However, using ^2^H NMR measurements, only a very small increase of acyl chain order was observed in the presence of 24*S*-HC [8]. Surprisingly, in the same study, 24*S*-HC and 27-HC exhibited a comparable effect on the acyl chain order compared to endogenous cholesterol measuring the diphenylhexatriene (DPH) anisotropy. Furthermore, high exchange rates of the molecules between erythrocytes and plasma were found [7], indicating that 24*S*-HC and 27-HC can – contrary to cholesterol – rapidly cross plasma membranes. Also for other oxysterols, like 7-ketocholesterol and 25-hydroxycholesterol, a lower tendency to form lateral lipid domains and an attenuated phospholipid condensation effect was found. These properties, which depend on the molecular position of the hydroxy group, were correlated with cytotoxic effects of the respective molecules [22–23].

In the current study, the influence of hydroxycholesterols on membrane properties such as lipid chain packing, membrane permeability, and membrane domain formation is investigated. These parameters are compared with those obtained for cholesterol by using various biophysical techniques such as NMR and fluorescence spectroscopy as well as fluorescence microscopy.

## Results

### Lipid chain order of hydroxycholesterol-containing membranes

**Figure 2:**
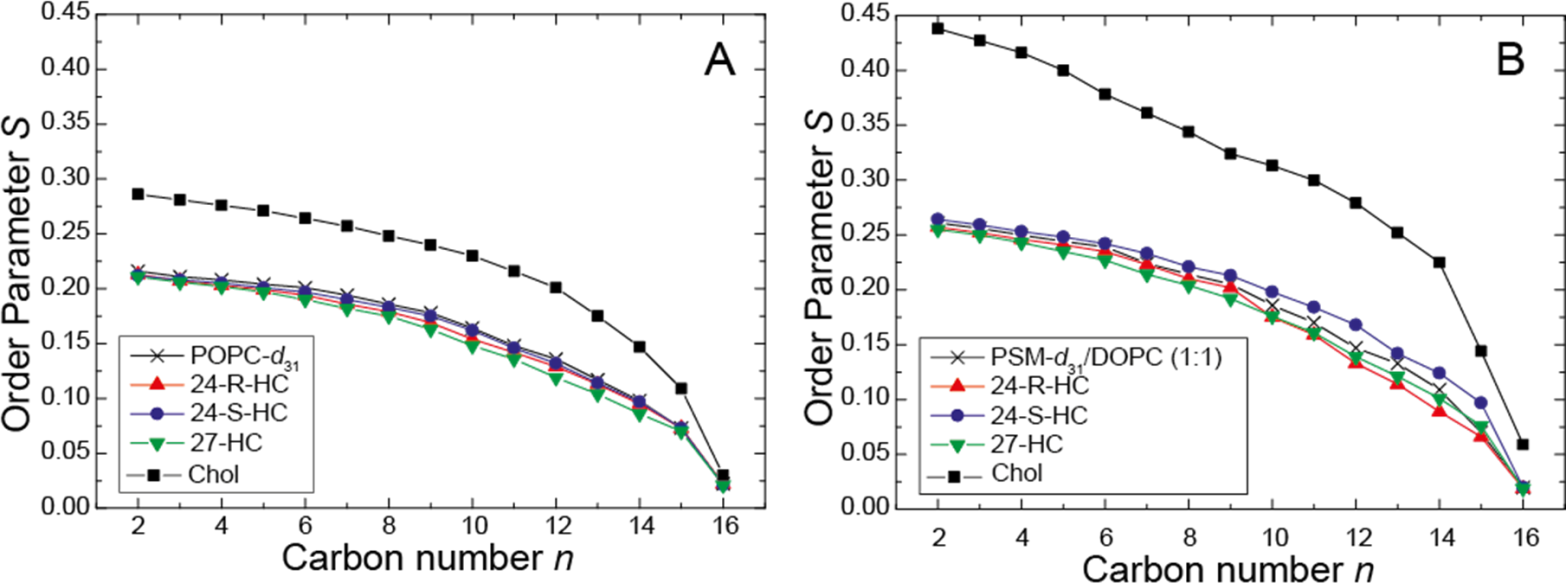
^2^H NMR chain order parameter of the *sn*-1 chain of (A) POPC-*d*_31_ in the absence and in the presence of the respective sterol (molar ratio 0.8:0.2) and (B) PSM-*d*_31_ in PSM-*d*_31_/DOPC/sterol membranes (molar ratio 1:1:1), (24*R*)-hydroxycholesterol (red), (24*S*)-hydroxycholesterol (blue) and 27-hydroxycholesterol (green) at a temperature of 30 °C. For comparison, the chain order parameters of a lipid membrane without any sterol and in the presence of the respective amount of cholesterol are shown in black [18]. Experimental errors are smaller than the symbol size.

The influence of the hydroxycholesterols on the lipid chain order and the degree of lipid condensation was investigated by ^2^H NMR measurements on lipid membranes of chain deuterated 1-palmitoyl-*d*_31_-2-oleoyl-*sn*-glycero-3-phosphocholine (POPC-*d*_31_) in the presence of 20 mol % of the respective hydroxysterols or cholesterol. The ^2^H NMR spectra (not shown) exhibited for all samples the typical superposition of Pake doublets with varying quadrupolar splittings as well-known for a lamellar bilayer membrane in the liquid-crystalline phase. From the ^2^H NMR spectra, chain order parameter plots were calculated, which are displayed in Figure 2A. As well-known from the literature, the presence of 20 mol % cholesterol leads to a pronounced increase in the 1-palmitoyl-2-oleoyl-*sn*-glycero-3-phosphocholine (POPC) chain order parameters [13,24]. In contrast, all three hydroxycholesterols did not induce such a cholesterol-like increase in POPC lipid chain order; in contrast, they caused a small decrease in lipid chain order compared with pure POPC membranes. While this decrease was insignificant for 24*S*-HC, it was more pronounced for 24*R*-HC, especially in the middle chain region, and quite substantial and out of the experimental error for 27-HC. These results are also reflected in the calculated lipid chain extent calculated using the mean torque model [25–26] (Table 1). Similar effects were observed in the lipid mixture, *N*-palmitoyl*-d*_31_-D-sphingomyelin (PSM-*d*_31_)/1,2-dioleoyl-*sn*-glycero-3-phosphocholine (DOPC)/hydroxycholesterol (molar ratio 1:1:1), which forms lateral membrane domains (Figure 2B), where all three hydroxycholesterols were not able to increase the lipid chain order parameters as observed for cholesterol. While for 24*S*-HC a very small increase compared to a pure lipid membrane without any cholesterol was observed, 24*R*-HC and 27-HC exhibited a decrease in lipid chain order again which was most pronounced in the middle chain region. Accordingly, the calculated lipid chain extents (Table 1) for the three hydroxycholesterols were close to the pure lipid membrane but significant smaller for a cholesterol containing membrane.

**Table 1:** The lipid chain extent of membranes consisting of POPC-*d*_31_ without and with the given cholesterols (molar ratio 0.8:0.2) and PSM-*d*_31_ in PSM-*d*_31_/DOPC/sterol membranes (molar ratio 1:1:1) were calculated using the mean torque model [25–26]. The values for pure POPC-*d*_31_ and in the presence of cholesterol are taken from the literature [19].

Sample	Chain extent [Å] for POPC-*d*_31_	Chain extent [Å] for PSM-*d*_31_/DOPC (1:1)

pure lipids	11.7 ± 0.1	12.3 ± 0.1
+ (24*R*)-hydroxycholesterol	11.5 ± 0.1	12.1 ± 0.2
+ (24*S*)-hydroxycholesterol	11.6 ± 0.1	12.6 ± 0.1
+ 27-hydroxycholesterol	11.3 ± 0.1	12.1 ± 0.1
+ cholesterol	13.2 ± 0.1	15.0 ± 0.2

### Influence of hydroxylcholesterols on membrane permeability

**Figure 3:**
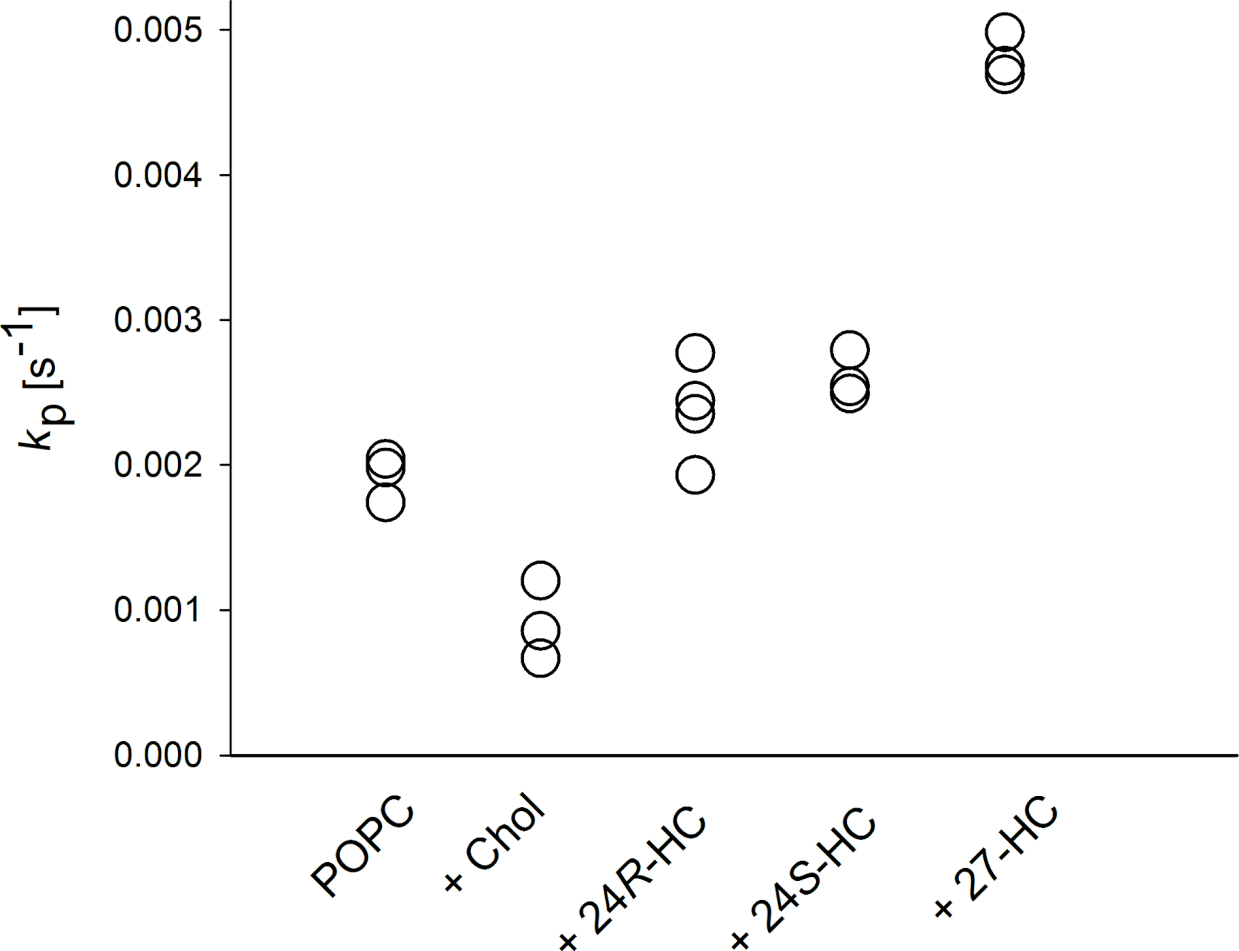
Rate constants for the permeation of dithionite across LUV membranes composed of POPC in the absence or in the presence of cholesterol or (24*R*)-hydroxycholesterol (24*R*-HC), (24*S*)-hydroxycholesterol (24*S*-HC) or 27-hydroxycholesterol (27-HC) (molar ratios 0.8:0.2) at 37 °C. All single values of the rate constants are shown which were determined from independent measurements.

The permeability of POPC membranes in the absence and in the presence of the respective sterol (molar ratio 0.8:0.2) was measured by using a fluorescence assay, which determines the permeation of dithionite ion across membranes (see Experimental, [18,27]). The rate constants of dithionite permeation in large unilamellar vesicles (LUVs) of varying lipid composition are shown in Figure 3 revealing that the rate constants of cholesterol-containing vesicles were lower than those of pure POPC LUVs. It is well-known that this sterol decreases the permeability toward polar molecules [28]. The rate constants in the presence of hydroxycholesterols were similar (24*R*-HC, 24*S*-HC) or even higher (27-HC) compared to those of pure POPC membranes indicating that these sterols are not able to seal a phospholipid membrane like endogenous cholesterol.

### Influence of hydroxycholesterols on the formation of lateral domains in giant unilamellar vesicles (GUVs)

**Figure 4:**
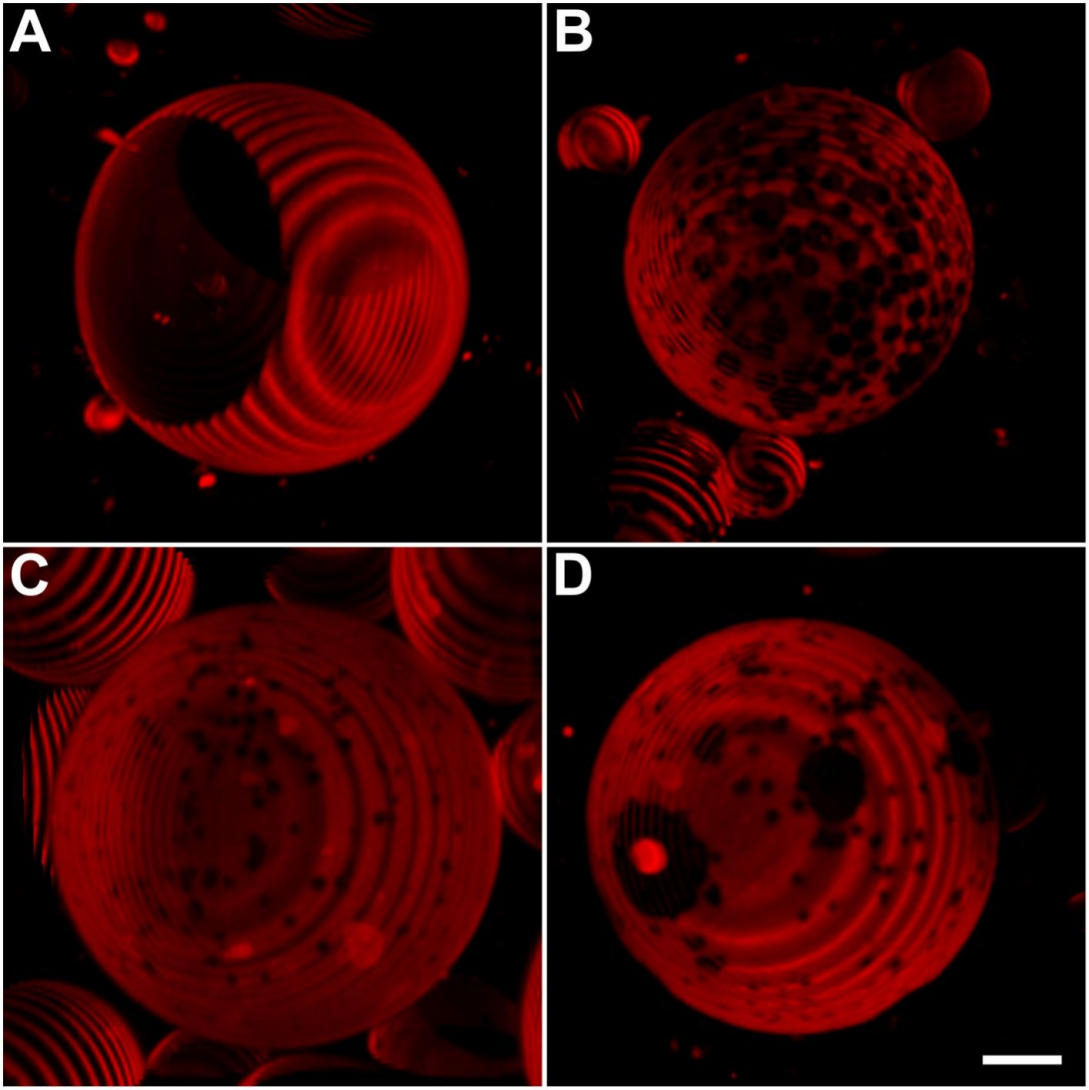
Confocal fluorescence images of GUVs containing DOPC/PSM/cholesterol (A), DOPC/PSM/24*R*-HC (B), DOPC/PSM/24*S*-HC (C), or DOPC/PSM/27-HC (D) (molar ratios 1:1:1). The GUV membranes were labeled with N-Rh-DOPE (0.5 mol %) that sorts preferentially into liquid disordered (ld) domains and z-stacks of the vesicles were recorded as described in the Experimental. Bar corresponds to 10 µm. The ring-like structures of the vesicles are caused by the assembly of the z-stacks having a step-size of 1 µm.

GUVs were prepared consisting of DOPC, PSM, and cholesterol at a molar ratio of 1:1:1. This lipid mixture is known for the formation and coexistence of lateral disordered (ld) and ordered (lo) domains. The domain structure was visualized by labeling the membrane with the ld marker 1,2-dioleoyl-*sn*-glycero-3-phosphoethanolamine-*N*-(lissamine rhodamine B sulfonyl) (ammonium salt) (N-Rh-DOPE) and recording z-stacks of the vesicles. The fluorescence microscopy images of cholesterol-containing GUVs show large membrane regions of low and of high fluorescence intensity, representing the lo and ld phase, respectively (Figure 4A). Note, that the vesicle shown in Figure 4A probably forms another dark lo domain on the back side. When cholesterol was substituted by hydroxycholesterols, the GUVs showed a different pattern of lateral domains (Figure 4B–D). These vesicles revealed a multitude of small ordered domains within the bright fluorescent disordered domains. We note that the GUVs shown in Figure 4 represent the majority of vesicles of the respective sample. A low percentage of vesicles were also observed having a different pattern of domains with respect to the shape and to the size.

## Discussion

Cholesterol is the main sterol of mammalian cell membranes and has a unique impact on membrane properties by that influencing important membrane functions. A special situation is encountered in the human brain, which contains a large amount of the body cholesterol. Almost all brain cholesterol is produced by local synthesis. It is the blood–brain barrier, which effectively protects the brain from the exchange with cholesterol provided from lipoproteins in the circulation, the cholesterol homeostasis in the brain is as far as possible self-contained. Regardless, a certain amount of cholesterol has to cross the blood–brain barrier in both directions. This transport function is fulfilled by hydroxycholesterols, which contain an additional hydroxy group in the alkyl chain compared to cholesterol, introducing a second polar moiety into the molecule rendering hydroxycholesterols more polar. It can be assumed that this modification alters the membrane properties of these molecules, which have been investigated so far only in a few studies (see Introduction). Therefore, the present study characterizes the impact of various hydroxycholesterols on membranes using various biophysical methods.

Our data show, that the influence of selected hydroxycholesterols on membrane properties differs from that of cholesterol with regard to (i) lipid chain order, (ii) membrane permeability and (iii) formation of lateral lipid domains.

Investigating their effect on the lipid chain order by ^2^H NMR, it was found that the three investigated hydroxycholesterols did not cause the typical lipid chain condensation mediated by endogenous cholesterol. Rather, some hydroxysterols caused a small decrease of lipid chain order. A similar tendency was observed for 24*S*-HC also using ^2^H NMR measurements [21]. This membrane behavior of the hydroxycholesterols can be rationalized in that the additional hydroxy moiety in these molecules diminishes their interaction with surrounding phospholipids. Such modifications can severely alter the orientation of a sterol in the membrane [29]. A similar situation has been encountered for estradiol, which also contains one hydroxy group on either end of the sterol [29]. In this work, a significant amount of the estradiol was found in the lipid water interface of the membrane with an orientation perpendicular to the membrane normal. By that, the molecules act disturbing rather than ordering. Notably, MD simulations found an opposite effect of the hydroxycholesterols [21]. The additional polarity of hydroxycholesterol also influences their impact on membrane permeability. For cholesterol, the induced membrane condensation causes a decreased penetration of polar molecules, e.g., water, across the membrane. This was proven here by measuring the permeation of dithionite across LUV membranes. The rate constant of dithionite permeation in POPC membranes containing cholesterol was reduced to about 50% compared to pure POPC vesicles. Replacing cholesterol by hydroxycholesterol, this effect was completely lost. The rate constants in the presence of 24*R*-HC and 24*S*-HC were similar to those of POPC membranes. For 27-HC, even an increase in permeation was observed, in these membranes dithionite permeated about 2.5 more rapidly than in pure POPC membranes. We note, that the changes of rate constants can also be explained by changes of lipid analogue flip-flop. However, in any case, they reflect membrane packing density towards the transbilayer permeation of a polar moiety.

As a third parameter, the impact of hydroxycholesterols on the formation of lateral membrane domains was investigated. Principally, these sterols are also able to trigger the formation of disordered and ordered domains, although they do not condense lipid chains as cholesterol does. Lipid condensation leads to thicker lo domains in membranes that are segregated from ld phase patches. However, it has also been shown that sterols that do not order lipid chains can induce lateral domain formation [14,30–31]. This can be rationalized by preferential interactions between sterols and saturated lipid chains, that represent the driving force for membrane domain formation even in the absence of a lipid condensation effect [32–33]. However, the pattern of domain formation was different with regard to their size and the number of domains per vesicle. This suggests that subtle differences in the interaction energies of the oxysterols and the other lipids of the mixture must exist. GUVs containing hydroxycholesterols show numerous small lo domains between the large ld domains. This indicates a different intermolecular interaction between the respective sterol and sphingomyelin being the basis for lipid segregation and, finally, domain formation.

What is the physiological consequence of the data presented? We could show that hydroxycholesterols at similarly large membrane concentrations like endogenous cholesterol do not disturb the bilayer structure of the membrane. Although, physiological membrane concentrations of hydroxycholesterols are not known, one can assume that these are much lower than those of cholesterol. However, due to the additional hydroxy group in the alkyl chain the interaction between the respective sterol and surrounding lipids (and proteins) is impacted. MD simulations for 27-HC have shown, that this molecule adopts compared to cholesterol different orientations within the membrane, which are upside-down, largely tilted and/or inter-leaflet positions [21].

These properties indicate that the molecules are less strongly anchored within the membrane bilayer, which guides their ability to cross the blood–brain barrier. This process consists of three steps at the membrane level, (i) incorporation of sterols into the plasma membrane, (ii) their transbilayer diffusion and (iii) their release from the membrane. With regard to the transfer of sterols to and from membranes, this can be principally realized via vesicular traffic or via monomeric transfer. The latter mechanism requires the presence of donors and acceptors, respectively, due to the low water solubility of sterols. Nevertheless, the import and export of cholesterol is rather slow [34], wherefore membrane proteins have been proposed to facilitate these processes. One putative function of those proteins could be to relieve the presentation of cholesterol molecules on the membrane surface for a better binding to extracellular acceptors (see [35]). It can be hypothesized, that the lower membrane embedding of hydroxycholesterols facilitates their membrane incorporation and/or release. Indeed, it was found that the transfer of hydroxycholesterols between erythrocytes and blood plasma is much faster than that of cholesterol [7].

With regard to the transbilayer mobility of sterols, it is generally assumed that cholesterol traverses the bilayer very rapidly by passive diffusion, although at certain conditions, e.g., special membrane compositions, its transbilayer movement could be compounded (see [35]). For hydroxycholesterols, the transbilayer movement has not been investigated so far. However, the studies measuring the transfer of those sterols between erythrocytes and plasma also indicate a rapid translocation of hydroxycholesterols across membranes [7].

## Conclusion

Our data show that, compared with endogenous cholesterol, hydroxycholesterols have a different influence on important membrane parameters which reflects an attenuated embedding of these sterols within the membrane.

## Experimental

### Materials

All lipids, POPC, DOPC, 1-palmitoyl-2-(12-[*N*-(7-nitrobenz-2-oxa-1,3-diazol-4-yl)amino]dodecanoyl]-*sn*-glycero-3-phosphocholine (NBD-PC), N-Rh-DOPE, POPC-*d*_31_, PSM-*d*_31_ as well as cholesterol and the three investigated hydroxycholesterols (structure see Figure 1) were purchased from Avanti Polar Lipids, Inc. (Alabaster, AL, USA). All other chemicals were purchased from Sigma-Aldrich (Taufkirchen, Germany) and were used without further purification.

### Preparation of NMR samples

The respective amounts of hydroxycholesterols and phospholipids were dissolved in chloroform at the respective molar ratios. The solvent was evaporated and the samples were re-dissolved in cyclohexane. After overnight lyophilization at high vacuum, the obtained fluffy powder was hydrated with 40 wt % deuterium-depleted water. The samples were equilibrated by ten freeze-thaw cycles and transferred into 5 mm glass vials and sealed.

### ^2^H NMR measurements

The ^2^H NMR experiments were performed on a Bruker DRX300 NMR spectrometer (Bruker BioSpin, Rheinstetten, Germany) at a resonance frequency of 46.1 MHz for ^2^H using a solid probe with a 5 mm solenoid coil. ^2^H NMR spectra were acquired using a quadrupolar echo pulse sequence [36] with a relaxation delay of 1 s. The two π/2 pulses with a typical length of around 3.2 µs were separated by a 50 µs delay. The spectral width was 500 kHz. ^2^H NMR spectra were dePaked and smoothed order parameters were determined as described in [37]. From these order parameters, the lipid chain extent was calculated according the mean torque model [25–26].

### Preparation of LUVs

LUVs were prepared using the extrusion method [38]. Aliquots of lipids dissolved in chloroform were combined in a glass vial and the solvent was evaporated in a rotating round-bottom flask under vacuum. Lipids were resuspended in a small volume of ethanol (final ethanol concentration was below 1% (v/v)), followed by the addition of HBS (HEPES buffered saline, 145 mM NaCl and 10 mM Hepes, pH 7.4, final lipid concentration 1 mM) and the mixture was vortexed. To prepare LUVs, this suspension was subjected to five freeze-thaw cycles followed by extrusion of the lipid suspension 10 times through 0.1 μm polycarbonate filters at 40 °C (extruder from Lipex Biomembranes Inc., Vancouver, Canada; filters from Costar, Nucleopore, Tübingen, Germany).

### Preparation of GUVs

GUVs were prepared using the electroswelling method [39]. Lipid mixtures were prepared from stock solutions in chloroform. Finally, 100 nmol of the domain forming lipid mixture of DOPC, PSM and cholesterol or the respective hydroxycholesterol (1:1:1, molar ratio) including 0.5 mol % of the liquid disordered (ld) domain marker N-Rh-DOPE were dissolved in chloroform and spotted onto custom-built titan chambers. These were placed on a heater plate at 50 °C to facilitate solvent evaporation, and subsequently subjected to high vacuum for at least 1 h for evaporation of remaining traces of the solvent. Lipid-coated slides were assembled using a spacer of Parafilm (Pechiney Plastic Packaging, Chicago, IL, USA) for insulation. The electroswelling chamber was filled with 1 mL sucrose buffer (250 mM sucrose, 15 mM NaN_3_, osmolarity of 280 mOsm/kg) and sealed with plasticine. An alternating electrical field of 10 Hz rising from 0.02 V to 1.1 V in the first 56 min was applied for 3 h at 55 °C.

### Permeation assay

For characterizing the permeation of polar molecules across the lipid membrane, an assay was applied which measures the transmembrane diffusion of dithionite [18–19,27]. The assay was performed in a similar manner to the procedure described in [19]. Briefly, LUVs containing POPC and 0.5 mol % NBD-PC without or with cholesterol or the respective hydroxycholesterol (molar ratio 0.8:0.2) were prepared. The NBD fluorescence intensity of 33 μM LUVs was recorded in a cuvette at 540 nm (λ_ex_ = 470 nm, slit width for excitation and emission 4 nm) at 37 °C using an Aminco Bowman Series 2 spectrofluorometer (Urbana, IL). After 30 s, sodium dithionite was added from a 1 M stock solution in 100 mM Tris (pH 10.0) to give a final concentration of 50 mM. Dithionite ions rapidly quench the fluorescence of the lipid analogs localized in the outer leaflet, which is reflected by a rapid initial decrease of fluorescence intensity (kinetics not shown). Subsequently, the fluorescence intensity decreased slowly caused by a slow permeation of dithionite ions across the bilayer. By that process, dithionite reacted with the NBD-PC molecules in the inner leaflet. After 300 s, Triton X-100 (0.5% (w/v) final concentration) was added, enabling complete reaction of dithionite with NBD-PC, resulting in a complete loss of fluorescence. The curves were normalized to the fluorescence intensities before addition of dithionite and were fitted to a bi-exponential equation. From the fittings, the rate constants for the rapid fluorescence decrease (representing reduction of NBD-PC in the outer leaflet) and those for the slow decrease (representing permeation of dithionite across the bilayer) were determined. The latter ones were used as the parameter for membrane permeability.

### Confocal laser scanning microscopy

For microscopy, a Visitron VisiScope scanning disk confocal laser microscope (Visitron Systems, Puchheim, Germany) with a 60× oil objective and an Andor iXon 888 EMCCD camera (1024 × 1024 pixels, Andor, Belfast, Northern Ireland) were used. N-Rh-DOPE was excited by a 561 nm diode laser.

Fife µL GUVs were mixed with 15 µL 250 mM glucose buffer (5.8 mM NaH_2_PO_4_, 5.8 mM Na_2_HPO_4_, osmolarity of 300 mOsm/kg, pH 7.2) in tissue culture treated microscopy suitable plastic dishes (ibiTreat µ-Slides Angiogenesis, ibidi, Martinsried, Germany). Vesicles were allowed to settle down some minutes before acquisition of z-stacks with 1 µm step size.
